# FOXM1 induces therapy resistance and inhibits apoptosis in a variety of human cancers

**DOI:** 10.1038/s41419-025-08321-5

**Published:** 2026-02-20

**Authors:** Sanjeev Raghuwanshi, Andrei L. Gartel

**Affiliations:** Department of Medicine, University of Illinois at Chicago, Chicago, IL USA

**Keywords:** Oncogenes, Translational research

## Abstract

FOXM1 (forkhead box protein M1) is a member of the transcription factors (TF) in the forkhead (FOX) family. Numerous studies over the past several years have progressively contributed to our current understanding of FOXM1 functions. Early work characterized FOXM1 as a proliferation-associated mammalian TF that controls cell cycle-transcriptional program, and is essential for proper mitotic function and genomic stability in normal cells. However, FOXM1 is aberrantly high-expressed in the majority of human cancers. A large body of literature from different studies has demonstrated FOXM1 as a critical molecule that regulates multiple aspects of cancer cells and maintains all major cancer hallmarks. In addition, recent studies have documented FOXM1 in cancer therapy resistance. Indeed, FOXM1 is repeatedly identified as a common factor associated with the higher cancer stage and weaker response to cancer therapies by regulating several targets relevant to drug response and cell survival. FOXM1-dependent transcriptional activity and downstream pathways regulate multiple functions in response to drug-induced genotoxic stress, oxidative stress, and mitotic catastrophe. FOXM1 also interacts with other proteins, and these protein-protein interactions regulate different functions and signaling pathways in response to drug-induced toxicity. Here, we mainly review and discuss our current molecular understanding of the mechanisms through which FOXM1 in cancer cells executes these new roles, and thereby induces therapy resistance and inhibits apoptosis in a variety of human cancers. We also discuss the opportunity and challenges for therapeutically targeting FOXM1 to induce apoptosis in drug-resistant cancers.

## Facts

- FOXM1 is overexpressed in the majority of human cancers and contributes to all the hallmarks of cancer.
- FOXM1 is repeatedly identified as a common factor associated with higher cancer stage and weaker response to cancer therapies.
- The microarray and pathway analysis demonstrated that FOXM1 controls a wide variety of cellular processes in cancers via regulating a plethora of transcriptional targets in the network.
- FOXM1-dependent transcriptional activity and associated pathways induce therapy resistance and inhibit apoptosis in a variety of human cancers.
- FOXM1 regulates multiple functions in response to drug-induced genotoxic stress, oxidative stress, and mitotic catastrophe.
- FOXM1 contributes to venetoclax resistance, in part through the upregulation of BCL2A1, an anti-apoptotic member of the BCL2 family.
- FOXM1 inhibitors have great synergy with different traditional chemotherapies and targeted cancer therapies.

## Open questions

- Can small-molecule FOXM1 inhibitors be used clinically as anticancer treatment?
- Can FOXM1inhibitors be more effective in the clinic when used in combination with existing therapies?
- Is FOXM1 inhibition during short-term treatment toxic to normal, proliferating cell compartments of the adult?
- Why have FOXM1 inhibitors not yet entered clinical trials? Is there a need for more thorough preclinical studies on their anti-cancer efficacy?

## Introduction

Cancer is a heterogeneous group of more than 100 diseases characterized by the uncontrollable growth of abnormal cells with the potential to spread to other parts of the body. The cancer burden is rising worldwide. Based on current estimates, it is the second-leading cause of mortality worldwide; the current report on global cancer statistics indicates 20 million new cancer cases and 9.7 million cancer deaths for the year 2022 [1]. Cancer is a major class of diseases with the most devastating effects, affecting the health of all human societies. Unfortunately, cancer is a dynamic and evolving disease. During the course of the disease, cancer becomes more heterogeneous, both molecularly and clinically [2]. This heterogeneity is a major challenge for its diagnosis and limits the efficacy of treatment.

Currently, chemotherapy is the most common therapeutic approach for various tumors across all stages of development to reduce cancer deaths [3]. Molecular targeted therapies and immune-based checkpoint therapies have also been evaluated in different preclinical and clinical trials [4, 5]. So far, many different classes of anticancer drugs have been approved by the FDA and are already being used in clinics. However, despite significant advancements in cancer research and therapy, the treatment options for most cancer patients are limited due to the intrinsic insensitivity or adaptive resistance to several therapeutic agents [6, 7]. Drug resistance continues to be the biggest challenge in the present therapeutic era.

The mechanisms of resistance to cancer drugs are diverse, leading pathways are complex and ill-defined. While cancer cells can develop resistance to traditional and targeted cancer therapies through a variety of mechanisms, FOXM1, a member of the forkhead (FOX) family of transcription factors (TF), has been repeatedly identified as a key factor associated with decreased response to different anti-cancer drugs. Several studies have revealed that FOXM1 overexpression is widespread in human cancers and associated with poor survival of cancer patients. A meta-analysis of 23 studies on human solid cancers found that FOXM1 expression is closely associated with worse 3-year overall survival and disease-free survival. FOXM1 overexpression was also linked to poor prognosis and advanced tumor stage in most solid cancers [8]. A cancer-wide meta-analysis using PRECOG also identified the FOXM1-regulatory network as a major predictor of adverse survival outcomes [9]. A recent study from our lab using the OHSU Beat AML database also identified FOXM1-transcriptional signatures with independent prognostic significance; low FOXM1 activity was identified as an independent predictor of chemotherapy response and disease-free survival of AML patients [10]. FOXM1 is a promising and attractive target for therapies in solid tumors and blood cancers.

FOXM1 primarily functions as a transcriptional activator. However, unlike other FOX family TFs, FOXM1 appears to bind to the forkhead consensus site (FKH) RYAAAYA (R = A/G, Y = C/T) with a relatively low affinity [11]. Early studies using coupled pull-down and gel-shift assays characterized the DNA sequence specificity and showed that FOXM1 prefers tandem repeats of a consensus site, “TAAACA” [12, 13]. Later biochemical and transcriptional activity analysis study using the isolated FOXM1-DBD did not show a clear preference for tandem consensus sites over the single site recognition in DNA-binding [11]. However, the results of a recent study illustrate that FOXM1 prefers to bind tandem sites to enable cooperative DNA recognition [14]. This cooperative DNA recognition may further confer specificity by enhancing the DNA-binding ability during transcriptional regulation. Current literature suggests that the mechanism by which FOXM1 interacts with DNA and transcription machinery for the regulation of its target genes is more complex. Several studies have mapped FOXM1 binding to the promoter regions and present conflicting models of FOXM1 recruitment. For example, Sadasivam et al. [15] using HeLa cells found that FOXM1 consensus sites were significantly enriched in genomic regions targeted by B-Myb and LIN9, which were predominantly located within cell cycle promoters. These data suggest the direct binding of FOXM1 to the FKH consensus, which is co-bound by the MuvB complex and B-Myb [15]. In contrast, Chen et al. [16] found no clear enrichment of FKH sites within FOXM1 binding regions in U2OS cells. This study identified just 270 binding regions for FOXM1; the majority of these regions were located in close proximity to the cell cycle gene promoters, and the majority of these sites overlapped with the B-Myb and LIN9 binding sites [16] uncovered by Sadasivam et al. [15]. This study suggests that FOXM1 binds to the chromatin regions through an alternative mechanism that involves the direct binding of FOXM1 to the MuvB/B-Myb complex rather than at the FKH consensus [16]. Another study using ChIP-seq analysis of FOXM1 binding in MCF7 and MDA-MB-231 cells [17] appears to support the existence of both models of recruitment. The data from Sanders et al. [18] support the findings of Chen et al. [16], who also identified FOXM1 binding regions lacking the FKH motif, where FOXM1 recruitment involved the direct interaction of FOXM1 with the MuvB complex. This study also demonstrates that FOXM1-DBD is necessary for the recruitment of FOXM1 to the DNA at FKH consensus and non-consensus binding sites and transcriptional activation [18]. They identified that the FOXM1-DBD mutants were unable to bind DNA yet maintained interactions with proteins like MuvB components, similar to the wild-type FOXM1 [18]. This study suggests three alternative models of FOXM1 recruitment to the chromatin: (1) FOXM1 directly binds at FKH consensus sites on gene promoters and interacts with muvB/B-Myb; (2) FOXM1 is recruited on gene promoters by MuvB complex and does not directly interact with DNA; (3) FOXM1 binds directly at non-consensus sites facilitated by interactions with muvB and B-Myb. A recent study using ChIP-Seq data from five cancer cell lines of different origins found the NFY binding motif at 81% of common FOXM1 binding sites in cell cycle-related gene promoters [19]. They suggest that FOXM1 can interact directly or indirectly through interaction with the NFY complex to regulate its cell cycle- and mitosis-related gene targets. They also proposed additional mechanisms, such as binding to super-enhancers and interacting with cell-type-specific TFs [19]. These data suggest that the primary function of FOXM1 as a transcriptional activator is more complex; it can regulate the expression of target genes by direct or indirect transcriptional activation involving protein-protein interactions with different cell-type-specific TF.

Typically, FOXM1 functions by the transcriptional activation of its targets. Several proteins, e.g., nucleophosmin (NPM), maternal embryonic leucine-zipper kinase (MELK), Polo-like kinase 1 (PLK1), Peptidylprolyl Cis/Trans Isomerase, NIMA-Interacting 1 (NPM1), STAT3, NF-κB (p65) protein, and noncoding RNAs such as LncRNA-PVT1, interact with FOXM1 and regulate FOXM1 expression, localization, and activity in cancer cells (reviewed in [20]). However, FOXM1 may also exert its oncogenic role by interacting with other proteins, such as β-catenin, SMAD3, DVL2, or PLK1. FOXM1 directly interacts with β-catenin [21, 22] or DVL2 [23] and promotes their nuclear localization and transcriptional activity in cancer cells. FOXM1-β-catenin interactions enhance the WNT pathway via enhancing Wnt target gene expression [21, 22]. FOXM1-DVL2 interaction in colorectal cancer (CRC) induces FOXM1/DVL2/Snail pathway through increasing the transcriptional activity of Wnt/β-catenin [23]. FOXM1 also interacts with SMAD3 in the nucleus and stabilizes the SMAD3/SMAD4 complex activity in breast cancer cells [24].

FOXM1, a master regulator of gene expression, directly or indirectly regulates a plethora of genes, and thereby controls a wide variety of cellular processes, including mitotic progression, cell growth and survival, DNA damage repair, redox signaling, cell migration, metabolism, senescence, and apoptosis [25, 26]. FOXM1 dysregulation is generally associated with the hallmarks of cancer; in current literature, FOXM1 is being frequently documented by various research groups for its role in cancer drug resistance. FOXM1 has been documented for drug resistance to a broad range of cancer therapies [10, 27], including genotoxic agents, microtubule inhibitors, and different targeted cancer therapies such as venetoclax. Most of these cancer therapies rely on the induction of DNA damage, mitotic catastrophe, and inhibiting anti-apoptotic proteins and other pro-survival pathways. Therefore, it is critical to uncover the molecular targets of FOXM1 and understand the mechanisms governed by FOXM1 in drug resistance to design more precise and efficient therapeutic approaches. In this review, we summarize findings on FOXM1-driven molecular mechanisms in drug resistance in multiple cancers. We also discuss the opportunity and challenges for therapeutically targeting FOXM1 to induce apoptosis in drug-resistant cancers.

## FOXM1 gene network promotes resistance to cell death induced by genotoxic agents

Deoxyribonucleic acid (DNA) is a very complex organic molecule that carries genetic information in our cells. It is also intrinsically a very reactive and highly sensitive molecule to the chemical modifications by different endogenous or exogenous agents. Indeed, DNA damage is common; human cells encounter tens of thousands of molecular lesions per day [28]. Fortunately, cells of the human body are equipped with a sophisticated and highly evolved DNA repair system, cell-cycle checkpoints, and death pathways, which allow cells to maintain the integrity of DNA and proper function. In most cases, our DNA is the target of various environmental genotoxic agents; DNA is also a main target of a class of anticancer drugs, which act by exerting DNA damage. Commonly used cytotoxic chemotherapies include platinum-based agents (e.g., cisplatin, carboplatin, and oxaliplatin) that inhibit DNA synthesis and function by cross-linking DNA strands [29], 5-Fluorouracil (5-FU) that damages DNA by interfering with thymidylate synthase (TS) enzymatic activity and incorporation of its metabolites into DNA and RNA [30], and inhibitors of topoisomerase (Top) 1 (such as the belotecan, topotecan, and irinotecan) [31] and Top 2 (such as the doxorubicin, epirubicin, and etoposide) [32] that interfere with the function of DNA by intercalating into DNA and generating Top-DNA adducts and DNA-strand breaks [31, 32], ultimately leading to tumor cell death. Cytotoxic chemotherapy is a cornerstone for the management of various malignancies across all stages of development; however, intrinsic or acquired resistance mechanisms limit the efficacy of different cytotoxic agents and lead to refractory and relapsed tumors. While resistance to cytotoxic agents is often a multifactorial process, FOXM1 expression and its activity have been observed in the majority of genotoxic drug-resistant human cancers (Tables 1–3).

**Table 1:** FOXM1-mediated mechanisms of resistance to various platinum-based drugs and alkylating agents.

SN	Cancer	Drug	FOXM1 role	Targets	Mechanisms of resistance	Ref
**Platinum-based drugs**						
1	Breast cancer	Cisplatin	FOXM1 up-regulation confers cisplatin resistance in MCF-7-CIS^R^ cells	DDR genes	Enhanced DNA-damage repair pathways.	[146]
2	NSCLC	Cisplatin	FoxM1 expression is associated with cisplatin resistance and poor prognosis in NSCLC patients.	-	Not discussed	[147]
3	Nasopharyngeal carcinoma (NPC)	Cisplatin	FOXM1 silencing enhanced the sensitivity of NPC cells to cisplatin.	NBS1	MRN (MRE11-RAD50-NBS1)-ATM axis regulation through NBS1.	[148]
4	Ovarian cancer	Cisplatin	Up-regulation confers cisplatin resistance in Ovarian cancer (A2780 and SKOV3) cells.	EXO1	Enhanced DNA-damage repair via regulation of EXO1.	[149]
5	Esophageal cancer Breast cancer	Cisplatin	Up-regulation confers cisplatin resistance in Esophageal cancer cells (FLO-1) and Breast cancer (HCC1143) cells.	-	FoxM1-dependent transcriptional activity and its downstream pathways.	[27]
6	Epithelial ovarian carcinoma (EOC)	Cisplatin Paclitaxel	Overexpression is associated with disease progression in serous type EOC patients. Up-regulation confers resistance in both cisplatin-resistant and paclitaxel-resistant IGROV1 cells. FOXM1 promotes EMT phenotype and CSCs formation.	-	FOXM1 and WNT/β-CATENIN pathway (FOXM1 interacts with β-CATENIN and facilitates its nuclear translocation and activation). Impairing cisplatin uptake via inhibiting hCTR1 and its regulatory transcription factor SP1.	[22]
7	Small cell lung cancer (SCLC)	Cisplatin	High FOXM1 expression is strongly associated with poor clinical outcomes of SCLC patients. FoxM1 silencing enhanced the sensitivity of cisplatin and etoposide-induced cytotoxicity in SCLC cells.	-	Not discussed	[150]
8	ovarian cancer (OC)	Cisplatin	FOXM1-mediated regulation of PCNA-associated factor P15^PAF^ promotes cisplatin resistance in OC cells.	P15^PAF^	PI3K/AKT/mTOR pathway activated by FOXM1/PCNA-associated factor P15^PAF^.	[151]
9	Retinoblastoma (RB)	Carboplatin	Up-regulation enhanced carboplatin resistance in Y-79CR cells.	ABCC4	FoxM1-ABCC4 axis	[152]
10	High-grade serous carcinoma (HGSC)	PARPi olaparib, Carboplatin	FOXM1 promote Olaparib and Carboplatin resistance in OVCAR8 cells.	-	DNA-damage repair pathway, homologous recombination (HR)	[153]
11	Colorectal cancer (CRC)	Oxaliplatin Vincristine	Overexpression confers oxaliplatin (L-OHP) and vincristine (VCR) resistance in HCT-8/L-OHP and HCT-8/VCR cells, respectively.	-	FOXM1/DVL2/Snail axis, FOXM1 interacts with DVL2 and facilitates its nuclear translocation.	[23]
12	Gastric cancer	Oxaliplatin Cisplatin	FOXM1/Mcl-1 enhancement is associated with poor prognosis of patients and decreased oxaliplatin-sensitivity in gastric cancer cells. FOXM1 suppression sensitized the gastric cancer cells to cisplatin as well as oxaliplatin.	Mcl-1	FOXM1/Mcl-1 pathway	[154, 155]
14	Non-serous epithelial ovarian cancer (EOC)	Cisplatin Carboplatin Doxorubicin olaparib.	FOXM1 overexpression was identified as an independent predictor of worse disease-specific survival in EOCs and of shorter time-to-progression in platinum-resistant cases. FOXM1 suppression enhanced the sensitivity of EOC cells to platinum, doxorubicin, and olaparib.	-	Deregulation of several FOXM1 putative targets with functions in cell cycle progression, DNA repair, metastasis, and drug response.	[58]
15	Advanced non-small cell lung cancer (NSCLC).	Cisplatin	Ectopic activation of the FOXM1-HMGA1-G6PD regulatory axis enhances the resistance of NSCLC cells to cisplatin and indicates a poor prognosis in NSCLC patients.	HMGA1	FOXM1-HMGA1-G6PD axis: promotes the pentose phosphate pathway, which enhances the supplies of NADPH and ribose for antagonizing cisplatin-induced ROS and DNA damage.	[156]
**Alkylating agents**						
16	Glioblastoma multiforme (GBM)	Temozolomide (TMZ)	TMZ-resistant recurrent GBM tumors and cell lines expressed higher levels of FoxM1. FoxM1 suppression inhibited Rad51 expression and sensitized recurrent GBM cells to TMZ cytotoxicity.	RAD51	FOXM1-RAD51 axis	[37]
17	High-grade glioma (HGG)	TMZ	FOXM1 was up-regulated in TMZ-insensitive glioma cells and HGG patients. FOXM1 suppression sensitized glioma to TMZ treatment (in vitro and in vivo).	Survivin	FOXM1-Survivin axis	[38]

**Table 2:** FOXM1-mediated mechanisms of resistance to anti-metabolites.

SN	Cancer	Drug	FOXM1 role	Targets	Mechanisms of resistance	Ref
1	Colorectal cancer (CRC)	5-FU	Upregulated in nonresponsive CRC patients and 5-FU-resistant CRC cells. Overexpression evokes 5-FU resistance in CRC.	ABCC10	FoxM1-ABCC4 axis (ABCC10, a multidrug resistance (MDR) protein that actively efflux drugs from cells)	[157]
2	CRC	5-FU	Elevated FOXM1/TYMS expressions promote acquired 5-FU resistance in colon cancer cells (HCT116 5-FU Res). Pharmacological targeting of FOXM1 restored 5-FU sensitivity.	TYMS and other 5-FU targets	Partially through the regulation of TYMS. FOXM1 targets such as DDR genes, matrix metalloproteinases, and cell cycle regulators might also play a role in 5-FU resistance.	[48]
4	Esophageal cancer and CRC	5-FU	Up-regulation confers 5-FU resistance in CRC and esophageal cancer cells. Pharmacological targeting of FOXM1 restored 5-FU sensitivity.	-	FoxM1-dependent transcriptional activity and its downstream pathways.	[27, 50]
5	Gastric Cancer stem-like cells (CSCs)	5-FU	FoxM1 is required for redox homeostasis and survival of gastric CSCs against chemotherapeutics.	Prx3	FoxM1-dependent expression of Prx3 was strongly associated with low levels of ROS and 5-FU resistance.	[158]
6	AML	AraC	Overexpression is associated with reduced efficacy of AraC therapy. FOXM1 silencing enhances the sensitivity of AML cells to AraC.	-	FOXM1-dependent transcriptional activity.	[10, 51, 52]

**Table 3:** FOXM1-mediated mechanisms of resistance to the inhibitors of topoisomerases.

SN	Cancer	Drug	FOXM1 role	Targets	Mechanisms of resistance	Ref
1	Triple-negative breast cancer (TNBC)	Doxorubicin (Dox)	FOXM1 expression is associated with a poor prognosis and higher recurrence rates after treatment. FOXM1 silencing in TNBC in vitro or with xenograft tumor sensitized the cells to Dox.	EXO1 RFC4 PLK4	FOXM1/NFκB1 interactions dependent regulation of DNA repair genes.	[159]
2	TNBC, Ovarian cancer, Esophageal cancer	Dox Irinotecan	Up-regulation confers resistance to topoisomerase inhibitors in ovarian cancer, TNBC, and esophageal cancer cells. Pharmacological targeting of FOXM1 restored Dox and irinotecan sensitivity.	-	FoxM1-dependent transcriptional activity and its downstream pathways	[27]
3	Human myeloma	Dox bortezomib (Bz)	Upregulation is common in relapsed multiple myeloma patients. High FOXM1 promotes Dox and Bz resistance. FOXM1 governs chromosomal instability and cell proliferation, also enhances drug efflux activity of myeloma cells.	-	FOXM1 genetic network and FOXM1-NEK2 and CDK4/6-Rb-E2F pathways	[160]
4	Breast cancer	Epirubicin	Overexpressed in the epirubicin-resistant cells. FOXM1 induces DNA damage-induced cellular senescence and resistance to epirubicin via regulating NBS1. FOXM1 suppression sensitized the proliferating cells of breast cancer and fibroblasts from mouse embryo (MEFs) to enter epirubicin-induced senescence.	NBS1	FOXM1-NBS1 axis	[60]
5	Bladder cancer	Dox	Overexpression of FOXM1 in bladder cancer cells (KU7 and 5637) increases drug efflux activity and Dox resistance.	ABCG2	FOXM1-ABCG2 axis (ABCG2, a MDR protein that actively efflux drugs from cells)	[161]
6	Epithelial squamous cell carcinoma	Dox	Squamous carcinoma cells use FOXM1 to control oxidative stress to escape premature senescence and apoptosis. FOXM1 suppression alone is sufficient to induce senescence in epithelial cells.	-	FOXM1-p63 positive feedback loop protects cells from oxidative stress-induced senescence and apoptotic cell death	[162]
7	Breast cancer	Dox Docetaxel	Co-expression of FOXM1, Survivin, and XIAP is associated with an unfavorable prognosis in patients. FOXM1 overexpression is associated with Dox and docetaxel resistance in breast cancer via regulating the expression of anti-apoptotic proteins, Survivin and XIAP.	Survivin and XIAP	FOXM1-Survivin and XIAP axis	[163]

### FOXM1-mediated mechanisms of resistance to cell death induced by platinum-based drugs and alkylating agents

Platinum-based chemotherapeutics, generally classified as alkylating (-Like) agents, including cisplatin, carboplatin, and oxaliplatin, have long been established in the routine treatment of ovarian, testicular, bladder, and colorectal cancer patients [33]. Nowadays, platinum-based compounds are largely used in the chemotherapy regimens for several cancer types; almost 50% of cancer patients receive a platinum-based chemotherapy regimen [34]. DNA is the major target of platinum-based drugs; treatment with these drugs generates monoadducts as well as DNA crosslinks that impair normal DNA function and induce apoptosis in tumor cells [34, 35]. However, cancer cells can develop resistance to cisplatin or its analogue drugs by activating a complex self-defense system primarily based on DNA repair by nucleotide excision and homologous recombination pathways [33, 35, 36]. Although the cisplatin resistance system can include several target genes that are activated or silenced in response to drug treatment, FOXM1 expression and its activity have been repeatedly associated with the progression of disease and resistance to the majority of platinum-based chemotherapies (Table 1). In different studies, FOXM1 expression has been correlated with the increased expression of different DDR proteins, likely increasing the efficiency of DNA damage repair pathways and maintaining cell survival. Besides DNA repair, FOXM1 also interacts with β-CATENIN and facilitates its nuclear transport and activation, promoting epithelial-mesenchymal transition (EMT), CSCs formation, and chemoresistance. FOXM1 was also reported to reduce the cytotoxic effect of platinum-based drugs via suppressing the hCTR1 (human copper transporter 1), that involved in transporting cisplatin into cells, and enhancing the ABCC4 (human ABC transporter) that pumps out platinum drugs. Moreover, recent studies have confirmed that FoxM1 may inhibit platinum drug-based apoptosis by up-regulating the pro-apoptotic Bcl-2 family protein Mcl-1. Several other FOXM1 targets have also been identified as the potential FOXM1-mediated resistance mechanisms to platinum-based chemotherapies (Table 1).

Temozolomide (TMZ) is a chemotherapeutic DNA alkylating agent that damages DNA by creating double-strand DNA breaks. It is widely used as a therapy for brain tumors such as high-grade gliomas. However, TMZ resistance is a major problem in the treatment and correlates with worse patient survival. Different studies have highlighted that TMZ-resistance is associated with increased FOXM1 levels [37, 38]. FOXM1 has been linked with TMZ-resistance via regulating two different mechanisms: one is enhanced DNA repair via RAD51 protein up-regulation in glioblastoma multiforme [37], and the second is the up-regulation of anti-apoptotic protein survivin in high-grade glioma [38]. Collectively, FOXM1 suppression with a combination therapy of thiostrepton or bortezomib with TMZ restored the sensitivity of cells to TMZ and increased apoptotic cell death.

The data discussed here suggest that FOXM1 induces resistance to platinum-based drugs and TMZ at different levels (Table 1); however, we and others have confirmed that genetic silencing of FOXM1 or pharmacological inhibition using FOXM1 inhibitors can restore the sensitivity of different malignancies to this group of drugs.

### FOXM1-mediated resistance mechanisms to cell death induced by anti-metabolites

Fluorouracil (5-FU), which belongs to a class of drugs called anti-metabolites, is a cytotoxic chemotherapy drug widely used in the treatment of several human cancers, including colorectal and breast cancer. 5-FU elicits cytotoxicity to cancer cells by interfering with essential biosynthetic processes, through inhibiting nucleotide synthesis enzyme thymidylate synthase (TS) enzymatic activity, as well as by misincorporating its metabolites (fluoronucleotides) into RNA and DNA [30, 39]. 5-FU is an analogue of uracil, in which the hydrogen atom at carbon position-5 (C-5) is replaced by a fluorine atom [30]. After administration in patients, more than 80% of it is primarily eliminated in the liver into pharmacologically inactive metabolites [40]. Only less than 3% 5-FU enters the cell (utilizes the same facilitated-transport system as uracil), and it is then metabolized into several active metabolites, including fluorodeoxyuridine monophosphate, fluorouridine triphosphate, and fluorodeoxyuridine triphosphate [30, 40]. These active metabolites elicit cytotoxic effects by disrupting RNA synthesis and inhibiting TS activity. 5-FU therapy for the treatment of colorectal cancer (CRC) was approved by the US FDA in 1962 [41]. Since its approval, 5-FU is widely used alone and in combination with various other chemotherapeutic agents in the treatment of a variety of solid tissue cancers, e.g., cancers of digestive organs, cervix, breast, and head and neck cancers [40]. 5-FU is one of the main first-line drugs for cancer treatment [42]; today, 5-FU-based therapy is used to treat several of the most lethal malignancies, including CRC and PDAC (pancreatic ductal adenocarcinoma) [40]. 5-FU is the most important chemotherapeutic drug for the treatment of CRC; however, the response rate of 5-FU in advanced CRC is still only 10–15% [43]. Even when 5-FU is combined with other chemotherapies such as irinotecan or oxaliplatin or with targeted therapies, the rate of response does not exceed 40–60% [44–46]. Over the last two decades, with an increase in the knowledge of its mechanism of action, various treatment strategies have been used to increase its anticancer activity. Despite these advances, frequent resistance to 5-FU remains a very significant limitation in the clinical use of the therapies based on 5-FU. Emerging technologies, such as microarray-based profiling of gene expression, are playing a significant role in identifying novel target genes and signaling pathways involved in mediating the resistance to 5-FU. The 5-FU-based therapy resistance may develop because of deficient drug uptake, alterations in the molecular structure of targets, activation of DNA-damage repair pathways, and anti-apoptotic pathways [47]. In several recent studies, FOXM1 and its transcriptional network have also been linked with the growth of tumor cells and resistance to 5-FU-based therapies (Table 2). Two recent studies on 5-FU-resistant CRC and cholangiocarcinoma cells reported high levels of FOXM1 and its target thymidylate synthase (TYMS) [48, 49]. FOXM1 induced the 5-FU resistance partially by increasing the drug target TS through direct binding to the TYMS promoter region and inducing its expression [48]. In addition, FOXM1 also increases drug efflux through ABCC10 and cellular ROS via regulating redox homeostasis [Table 2]. We and others have demonstrated that FOXM1 transcriptional activity and its downstream pathways regulate a broad range of cellular processes, including DDR, redox signaling, and cell cycle progression [27, 48, 50], suggesting that FOXM1 can promote 5-FU resistance in cancer cells at various levels. However, in our recent studies, we found a very strong synergy between 5-FU and novel FOXM1 inhibitors STL001 [27].

Cytarabine (AraC) is another anti-neoplastic anti-metabolite, which is commonly used for the treatment of various blood cancers such as acute myeloid leukemia (AML). Cytarabine resistance in leukemia can arise from several different mechanisms, including FOXM1-dependent transcriptional activity. We have previously demonstrated that AraC treatment in AML increases FOXM1 levels [10, 51, 52]. FOXM1 overexpression in AML is associated with reduced efficacy of AraC therapy; however, FOXM1 suppression sensitized resistant cells to AraC therapy [10, 51, 52]. Indeed, FOXM1 inhibition using STL001 was very effective to overcome 5-FU resistance in solid cancers [27] and AraC resistance in AML [10]. Hence, pharmacological targeting of FOXM1 in combination with AraC therapy might be very effective in increasing the treatment efficacy.

### FOXM1-mediated mechanisms of resistance to topoisomerase inhibitors

DNA topoisomerases are essential enzymes that solve topological problems arising due to the double-helical structure of DNA. During DNA replication, transcription, recombination, and chromatin remodeling in cells, topoisomerases are involved in breaking and rejoining DNA molecules in a controlled manner that reduces torsional strain caused by the unwinding of the DNA double helix [53, 54]. Topoisomerases are generally classified into two categories: type 1 topoisomerases, which cleave a single strand of DNA, and type 2 topoisomerases, which cleave both strands of DNA [53, 54]. Due to their important role in the maintenance of DNA structure and topology integrity, DNA topoisomerases are the targets of important anticancer drugs, such as the inhibitors of Top 1 (irinotecan, belotecan, and topotecan) and Top 2 (doxorubicin, epirubicin, and etoposide) [31, 32]. This class of chemotherapy drugs exerts its cytotoxic effects by blocking topoisomerase activity and stabilizing topoisomerase-DNA cleavable complexes, leading to the formation of lethal DNA breaks that drive the cells into apoptosis [31]. The inhibitors of topoisomerases are approved for the treatment of a variety of malignancies, including colorectal, ovarian, breast, testicular, and small-cell lung cancers, as well as myeloid malignancies [55]. Although this class of molecules has sparked much attention in recent years because of their proven anticancer properties, resistance is still a critical clinical problem. Based on recent studies, reduced drug accumulation in tumor cells, alterations in the target protein, or alterations in the cellular response to the drug are among the several key contributing mechanisms of resistance [56, 57]. FOXM1 is upregulated in various cancer cells and recognized as one of the key factors in resistance to the inhibitors of topoisomerases [27, 58, 59]. The function of FOXM1 in resistance to the topoisomerases is associated with the increase in DDR proteins such as ATM [10], NBS1 [60], and others discussed in Table 3. It is also closely associated with an increase in anti-apoptotic genes such as *survivin* and *XIAP*, and MDR proteins such as ABCG2 (Table 3). We and others have found that FOXM1 transcriptional activity and its downstream pathways may affect several biological processes, such as DNA-damage repair, cell-cycle regulation, oxidative stress, senescence, and apoptosis [Table 3]. Different studies in our lab and others have demonstrated that FOXM1 suppression enhanced topoisomerase inhibitors-induced apoptosis in a variety of cancers (Table 3) [27, 61, 62].

## FOXM1-mediated mechanisms of resistance to cell death induced by taxanes

Paclitaxel (PTX) and docetaxel (DTX) are members of the taxane family and the most commonly used microtubule-stabilizing class of cancer drugs [63]. They have a similar mechanism of action, bind to β-tubulin subunit, and impair microtubule dynamics by inducing microtubule stabilization and preventing its depolymerization [63–65]. This affects mitosis and cytoskeleton functions, leading to cell cycle arrests at G2/M phase, and suppresses proliferation and viability of cancer cells [63]. While taxanes are well known for their mitotic activity, they can also possess non-mitotic functions by affecting various molecular pathways and cellular processes [66]. Taxanes are well-known chemotherapeutic agents, used both as monotherapy and in combination with other agents like doxorubicin and cisplatin as first or second treatment options in various treatment regimens [65–67]. Taxanes, like PTX and DTX, are used to treat various cancers, including breast, lung, and ovarian cancers [63]. However, cancer cells can develop resistance to taxanes, making these drugs less effective in treating cancer patients. Various molecular pathways in cancer cells are involved in mediating taxane resistance [68, 69]. We and others have reported that FOXM1 regulates a transcriptional network essential for mitotic cell division [27, 50, 70]. Mitotic defect and premature senescence in cells with FOXM1 deficiency were associated with the reduced expression of Cdc25B, Aurora B kinase, survivin, PLK1, CENPA, and CENPB [70]. In our recent study, genetic inhibition or pharmacological suppression of FOXM1 using STL001 (a selective FOXM1 inhibitor) was analysed using RNA-Seq, data revealed that the FOXM1-dependent transcriptional activity significantly affects cell cycle and mitotic checkpoint regulatory pathways (AURKB, E2F, and PLK1) [27]. FOXM1 suppression also sensitized prostate cancer cells to paclitaxel-induced apoptosis in vitro [27]. Several other reports also found FOXM1 as a main determinant of taxane resistance by regulating the formation of normal mitotic spindles, chromosome alignment, and segregation through up-regulating KIF20A (kinesin protein), Stathmin (microtubule-destabilizing protein), and KIF2C, which is a microtubule depolymerase (discussed in Table 4). FOXM1 also increases taxane resistance by increasing the activity of various molecular pathways, epigenetic regulators, and multidrug resistance (MDR) protein that actively efflux drugs from cells (Table 4). These studies indicate that FOXM1 contributes to taxanes resistance through diverse molecular mechanisms. However, we and others have identified that pharmacological targeting of FOXM1 synergized with the inhibitors of microtubule functions, such as taxanes and vincristine, and induced senescence and apoptotic cell death (Table 4) [27, 50, 71, 72]. Hence, FOXM1 targeting in combination therapies with taxanes or vincristine may have promising therapeutic benefits for the successful treatment of cancer.

**Table 4:** FOXM1-mediated mechanisms of resistance to taxanes-induced cell death.

SN	Cancer	Drug	FOXM1 role	Targets	mechanisms of resistance	Ref
1	Prostate cancer	Docetaxel	Overexpressed in docetaxel-resistant cancer cells (DU145-DR). FOXM1 controls cancer stem cells (CSCs) and contributes to taxanes resistance via regulating UHRF1.	Uhrf1	FOXM1-UHRF1 axis. (UHRF1 is a critical epigenetic regulator involved in therapeutic resistance)	[164]
2	Castration-resistant prostate cancer (CRPC)	Docetaxel	Upregulated in the docetaxel-resistant CRPC cell lines (PC3-DR and VCaP-DR). FOXM1 suppression sensitized the cells to docetaxel both in vitro and in vivo.	-	Autophagy pathway activated via FOXM1 targeted AMPK/mTOR pathway.	[165]
3	Gastric cancer	Docetaxel	Identified as an independent survival indicator in gastric cancer patients. Promotes docetaxel resistance by up-regulating the microtubule-destabilizing protein Stathmin.	Stathmin	FOXM1-Stathmin axis.	[166]
4	Human breast cancer	Paclitaxel Herceptin	Overexpression makes cancer cells resistant to Herceptin (HER2 receptor- targeted monoclonal antibody) and paclitaxel, both as single agents and in combination. FoxM1 expression is higher in resistant lines (SKBR3, MDA-MB-453 and BT474)	Stathmin	FOXM1-Stathmin axis promotes Taxol resistance. FOXM1 involved in herceptin resistance by preventing the accumulation of p27 that is required for herceptin induced G1/S arrest.	[167]
5	Human pancreatic cancer and	Paclitaxel	FOXM1 expression levels were stable in resistant cells when treated with paclitaxel. Overexpression of FoxM1b or FoxM1c conferred Panc-02 resistance to paclitaxel. Silencing either or both (FOXM1 and PHB1) of the molecules sensitized the resistant cell to paclitaxel in vitro and in vivo.	PHB1	FoxM1/PHB1/RAF-MEK-ERK feedback loop activated ABCA2 expression participates in paclitaxel resistance.	[168]
6	Breast cancer	Paclitaxel	Stable FoxM1 expression level in paclitaxel-resistant cell line (MCF-7 TaxR) contributes to resistance. Deletion of FOXM1 inhibits cell viability and induces mitotic catastrophe-dependent cellular senescence in response to paclitaxel.	KIF20A	FOXM1-KIF20A axis (KIF20A is a kinesin involved in mitotic progression)	[169]
7	Prostate cancer (PCa)	Docetaxel	Overexpression confers resistance to apoptosis induced by docetaxel in docetaxel-resistant PCa cell lines (DU145-DR and VCaP-DR). FOXM1 suppression contributes to the docetaxel sensitiveness in vitro and in vivo.	KIF20A	FOXM1-KIF20A axis.	[170]
8	Nasopharyngeal carcinoma	Paclitaxel	FOXM1 and ABCC5 expression were positively correlated in paclitaxel-resistant nasopharyngeal carcinoma cells and tumor tissues.	ABCC5	FOXM1-ABCC5 axis.	[171]
9	Ovarian cancer (OC)	Paclitaxel	FOXM1 expression as an independent prognostic factor for OC. FOXM1 overexpression confers paclitaxel resistance in OC cells. FOXM1 knockdown enhanced paclitaxel-mediated mitotic catastrophe and cell death in a p53-independent manner.	KIF2C	FOXM1-KIF2C axis. (KIF2C is a well-known microtubule depolymerase.)	[172]

## FOXM1-mediated resistance to targeted cancer therapies

Several recent studies have revealed that FOXM1 can directly and indirectly be a culprit in different targeted cancer therapies. FOXM1 overexpression mediates resistance to different targeted therapies and its suppression was found as a very effective strategy in sensitizing various cancers to different targeted cancer therapies, such as estrogen receptor antagonists (tamoxifen) therapy in receptor-positive (ER + ) breast cancers [73], endocrine therapy (anti-androgens) in prostate cancer [74, 75], reactive oxygen species (ROS) inducers (PEITC or 2-methoxyestradiol) in breast cancer [76], PARP inhibitors (Olaparib) in in pancreatic and triple-negative breast cancers [77, 78], angiogenesis inhibitors (VEGFR2 monoclonal antibodies) in hepatocellular carcinoma [79], and BCL2 inhibitor (Venetoclax) in AML [80]. The FOXM1-mediated resistance mechanisms to the above-discussed targeted therapies are often multifactorial or unknown. However, the combination of targeted cancer therapies and FOXM1 inhibitors is a promising therapeutic strategy in enhancing the anti-tumor activity and increasing patient survival.

### FOXM1-mediated mechanisms of resistance to BCL2 inhibitor, Venetoclax

Apoptosis can be induced by a range of extracellular (extrinsic) or intracellular (intrinsic) stimuli; in recent years, the molecular mechanisms regulating the apoptotic pathways have been extensively investigated [81]. The intrinsic apoptosis pathway relies on the balance between pro- and anti-apoptotic members of the BCL2 family, inducing mitochondrial outer membrane permeabilization (MOMP), leading to the release of cytochrome C and caspase-dependent cell death [81, 82]. Different BCL2 family members possess at least one of four relatively conserved sequence motifs called BCL2 homology (BH1–BH4) domains [82, 83]. The anti-apoptotic proteins, specifically BCL2, and its relatives (BCL2L1 [Bcl-xL], MCL1, BCL2L2 [BCL-w], and BCL2A1) promote cell survival by direct binding to pro-apoptotic proteins at their BH3 motifs and sequester them [82, 83]. The pro-apoptotic members of the BCL2 protein family instead elicit cell death and consist of BH3-only proteins and effector proteins. The effector proteins (BAK and BAX) have BH1-4 motifs; however, BH3-only proteins have a single BH3 domain, and they act as sensitizers (BAD, HRK, BIK, and NOXA) or activators (BID, BIM and PUMA) of apoptosis [81, 83]. These sensitizer BH3-only proteins lack the capacity to directly activate effectors (Bax and Bak). Rather, they act indirectly by displacing the BH3-only activators from BCL-2 anti-apoptotic proteins, thereby releasing the activators to trigger the activation of BAX and BAK [84]. Upon activation by bound BH3–only activator proteins, BAK/BAX protein oligomerizes, leading to MOMP and initiation of cytochrome c-mediated intrinsic apoptosis [83].

The augmentation of the anti-apoptotic BCL2 family proteins is one of the hallmarks of cancer [82]; dysregulation of these proteins contributes to cancer progression and drug resistance [85–87]. Targeting the specific anti-apoptotic protein of the BCL-2 family is a novel and promising therapeutic strategy. Navitoclax (ABT-263) was the first extensively studied BCL-2 and BCL-XL dual antagonist; this compound has demonstrated promising clinical activity in lymphoid malignancies and also enhanced docetaxel efficacy in preclinical studies with solid tumors. However, the incidences of thrombocytopenia and neutropenia caused by BCL-XL inhibition was the most commonly observed dose-limiting toxicity of navitoclax [82, 88]. Subsequently, a potent and highly selective BCL-2 inhibitor, venetoclax (ABT-199), was developed and approved by the US FDA for the treatment of leukemia. It is a promising small molecule currently in the clinic to treat patients with CLL (chronic lymphocytic leukemia), AML, and other hematologic malignancies [82, 89]. In the class of BH3 mimetics, venetoclax is a potent BCL2-selective BH3-mimetic that has shown substantial antitumor activity with durable responses in the majority of patients with previously treated CLL or small lymphocytic lymphoma, and therefore, venetoclax was approved by the FDA as an effective treatment of these diseases [90, 91]. For older patients with newly diagnosed (de novo) AML, the venetoclax in combination with low-dose hypomethylation agents induced ~70% response rates [92, 93]. However, venetoclax had modest activity in relapse/refractory AML patients as a single agent (CR/CRi rates 19%) or in combination with hypomethylation agents (CR/CRi rates 54%) [94, 95].

Like other therapies, patients who initially respond to venetoclax will ultimately relapse because of acquired resistance following venetoclax treatment. Less is known about the mechanism of resistance; diverse mechanisms have the potential to contribute to venetoclax resistance, but recent reports have highlighted a decrease in BCL2 dependence and a shift in dependencies on alternative anti-apoptotic BCL-2 family proteins [83, 96–101]. As we discussed earlier, FOXM1 expression is abnormally high in the majority of human cancers and is associated with a reduced response to therapy in several cancers. Recent studies on multiple myeloma have highlighted a synergy between FOXM1 inhibitors and venetoclax [102, 103] (Table 5). In earlier reports from our lab (Table 5), we have also determined that FOXM1 transcriptional activity is strongly associated with resistance to venetoclax-induced apoptosis, and knockdown or pharmacological targeting of FOXM1 sensitizes AML cells to venetoclax [10, 52]. A recent transcriptomic analysis of AML patients’ samples identified BCL2A1 as one of the major drivers affecting venetoclax sensitivity in AML [87]. Interestingly, our recent study using RNA-Seq and quantitative RT–PCR analysis identified BCL2A1 with the most intriguing pattern of expression changes associated with FOXM1 levels [80]. We identified that pharmacologic inhibition of FOXM1 with a selective FOXM1 inhibitor, STL001, or genetic suppression of FOXM1 reduced the expression of BCL2A1 and sensitized AML cells to venetoclax-induced apoptosis [80]. Further studies using genetic suppression or overexpression of FOXM1 and BCL2A1 spotlighted the correlation between FOXM1-induced and BCL2A1-induced resistance to venetoclax. We have shown that FOXM1-dependent resistance to venetoclax in AML is determined by BCL2A1 [80] (Table 5). Therefore, we proposed a novel combination of venetoclax with STL001 as a novel therapeutic approach for the treatment of venetoclax-resistant AML [80].

**Table 5:** FOXM1-mediated mechanisms of resistance to BCL2 inhibitor (Venetoclax).

SN	Cancer	Treatment	FOXM1 role	Targets	Mechanisms of resistance	Ref
1	AML	Venetoclax Cytarabine	FOXM1 transcriptional activity in AML patient samples can determine response to chemotherapy and independently predict disease-related death. FOXM1 inhibitions sensitize AML cell lines – including KG-1, HL-60, and K562 to chemotherapy and BCL2 inhibitor venetoclax.	-	Not discussed	[10]
2	Multiple myeloma (MM)	Venetoclax	Highly expressed in human MM cell lines (OPM2 and Delta47). Inhibiting FOXM1 sensitizes MM cells to venetoclax and prolongs the survival time of the OPM2-engrafted NSG mice. FOXM1 inhibitor and venetoclax have a synergistic effect in vivo and ex vivo.	-	Not discussed	[102, 103]
3	AML	Venetoclax	High activity of FOXM1-AKT loop promotes AML cell survival and resistance to venetoclax-induced apoptosis in AML cells.	-	FOXM1-AKT loop	[52]
4	AML	Venetoclax	Venetoclax-induced apoptosis is inhibited by FOXM1 in AML through upregulation of BCL2A1. Pharmacological targeting of FOXM1 or genetic suppression of BCL2A1 restored the sensitivity of AML cells to venetoclax-induced apoptosis.	BCL2A1	FOXM1–BCL2A1 axis	[80]

## FOXM1 role in cell cycle progression and drug resistance

FOXM1 plays an important role in both development and tumor progression by regulating a wide variety of cellular activities [104]. Among several functions, its role in cell cycle regulation is fundamental and has been well studied [70, 105–107]. During G1/S transition, Cyclin-Dependent Kinases 4 and 6 (Cdk4/Cdk6) stabilize and activate FOXM1 by phosphorylation at multiple sites [108]. Several cell-cycle genes involved in the regulation of G1/S and G2/M transition are targeted by FOXM1 [108–110]. During the G1/S transition, FOXM1 transcriptional activity is essential for the expression of Skp1-Cullin 1-F-box (SCF) ubiquitin ligase subunits SKP2 and CKS1, which target CDK-inhibitor (CDKI) proteins P21 and P27 for degradation, thereby promoting cell cycle S phase entry [70]. More reports highlight its functional role in G2/M transition and mitosis. During the late G2 phase of the cell cycle, FOXM1 is required for proper mitotic entry and progression by controlling the expression levels of a cluster of genes critical for the G2/M transition, such as CDC25B, AURORA B, CYCLIN B1, PLK1, and CENP F [70, 107, 110]. The transcriptional activity of FOXM1 during G2/M transition requires its activation by the Cdk1/Cdk2 kinases and PLK1 [107, 111, 112]. The PLK1-dependent phosphorylation of FOXM1 increases its transcriptional activity, reaching maximal stimulatory activity during G2/M phases, where Cyclin/Cdk kinase and Plk1 are expressed at high levels by FOXM1 [107, 111]. It is noteworthy that FOXM1 stimulates expression levels of the Cdc25b phosphatase and Cyclin B1 that activate Cdk1 [105]. Thus, FOXM1, Cdk1/Cdk2, and Plk1-derived feedforward loops activate each other in G2/M phases. At the exit of M phase and early G1, FOXM1 becomes dephosphorylated and degraded in a Ub-proteasome pathway involving APC/C-Cdh1 complex [113, 114]. FOXM1overexpression and its interactions with different cell cycle proteins are important for cancer growth and survival, rationalizing the use of FOXM1 inhibitors and cell cycle-based therapies in combination to synergistically enhance apoptosis in cancer cells.

### FOXM1-mediated resistance to Aurora Kinase A (AURKA) Inhibitors

Aurora Kinase A (AURKA) is a nuclear protein, has a significant role in promoting bipolar microtubule spindle assembly and proper cell division [115]. Alisertib is a specific AURKA inhibitor that has been tried in the treatment, but as a monotherapy, it failed to show better anti-cancer activity than other therapies [116]. The mechanisms of resistance to AURKA inhibitors are largely unknown. Recently, Yang et al. uncovered a positive AURKA-FOXM1 feedback loop that is crucial for the self-renewal and may be involved in drug resistance of breast cancer stem cells (BCSCs) [117]. They found that FOXM1 transcriptionally activates AURKA expression; on the other hand, AURKA activates FOXM1 expression by directly binding the FOXM1 promoter in a kinase-independent manner [117]. Accordingly, a synergistic activity of FOXM1 and AURKA inhibitors disrupts the AURKA/FOXM1-positive feedback loop, resulting in more effective inhibition of the tumorigenicity and self-renewal ability of BCSCs [117].

### FOXM1 as a resistance mechanism to Polo-like Kinase 1 (PLK1) Inhibitors

A main mitotic kinase, PLK1, is a therapeutic target for several cancers due to its crucial function in mitosis [107]. Numerous PLK1 inhibitors have been extensively studied in preclinical studies. Most of them have dose-limiting side effects and modest anti-cancer response seen in clinical trials [118]. PLK1 inhibitors in combination with other cancer therapies may be vital to combat issues observed with PLK1 inhibitors as monotherapy. A recent report found FOXM1 overexpression in diffuse large B-cell lymphoma (DLBCL) cancer cells treated with PLK1 inhibitors [119]. They revealed that the deregulated FOXM1-PLK1 axis mediates cell growth and survival in DLBCL [119], rationalizing the use of FOXM1 and PLK1 inhibitors in combination to synergistically enhance apoptosis in cancer cells. The combination of FOXM1 and PLK1 inhibitors was found to be effective against the growth of DLBCL cancer cells in vitro [119], also inhibited papillary thyroid carcinoma tumor growth in vivo [120]. Consequently, there is a strong rationale for dual-targeting PLK1 and FOXM1 in solid tumors where the FOXM1-PLK1 axis is activated.

### FOXM1 inhibitors and CDK4/6 inhibitors act synergistically

Genetic alterations in cell cycle regulatory genes and hyperactivity of the Cyclin D–CDK4/6 axis are common in cancers and are important for the growth and survival of many cancer types [121]. Blocking G1/S transition by targeting cell cycle proteins can be a successful strategy in cancer treatment. Since 2015, the FDA has approved three CDK4/6 inhibitors, palbociclib, ribociclib, and abemaciclib for hormone receptor-positive and HER2-negative advanced and metastatic breast cancers [122]. However, studies have shown that intrinsic or acquired resistance pathways can cause cells to be insensitive to CDK4/6 inhibitors [121, 122]. Therefore, most of the available clinical trials are investigating CDK4/6 inhibitors in combination with other anticancer agents. The CDK4/6 proteins in complex with cyclin D are critical mediators in facilitating the G1/S phase transition by phosphorylating and disassociating retinoblastoma (RB) protein from E2F TF [70]. CDK4/6 also phosphorylates FOXM1 at multiple sites and transcriptionally activates FOXM1 during the G1/S phase [108], providing a rationale for the combination of FOXM1 inhibitors with CDK4/6 inhibitors [121]. Indeed, a recent report by Guillen et al. [123] showed that CDK4/6 and FOXM1 inhibitors work synergistically and enhance the growth suppression of ER-positive breast cancer cells.

## Pharmacologic inhibition of FOXM1

Many labs, including ours, have confirmed that high FOXM1 levels are generally associated with enhanced drug resistance of cancer cells. There is strong rationale for targeting FOXM1 in cancer, and therefore, there is an increasing interest in finding small-molecule inhibitors of FOXM1 (Table 6). Microbial-derived thiazole antibiotics/proteasome inhibitors siomycin A and thiostrepton are the first reported inhibitors of the transcriptional activity of FOXM1. Although thiostrepton was shown to interact directly with FOXM1 [124], it is a broad-spectrum proteosome inhibitor, which makes its FOXM1 selectivity uncertain [125, 126]. Several reports have shown that the thiostrepton sensitizes cancer cells to chemotherapies [126, 127]; however, the mechanistic link between these phenotypic effects and FOXM1 is uncertain. The heterocycle FDI-6 is a small molecule FOXM1 inhibitor, discovered in a high-throughput screening of small molecules; it inhibits FOXM1-DNA binding and disrupts the transcription of FOXM1-activated genes [128]. Although FDI-6 has shown anticancer effects tested in different in vitro and in vivo studies [126, 129], it does not show adequate in vivo pharmacokinetic (PK) properties [129, 130]. The poor PK limits its effectiveness as a cancer therapeutic agent. A novel membrane-transducing FOXM1-inhibiting p19ARF26-44 peptide containing nine D-Arg residues to enhance cellular uptake of the peptide has been used effectively in preclinical cancer treatment studies [73, 131, 132]. The FOXM1-inhibiting peptide binds to FOXM1 with the help of ARF sequences and localizes it to the nucleolus with the help of Arg-rich sequences [131]. Although p19ARF26-44 peptides are effective in inhibiting FOXM1 transcriptional activity and the growth of cancer cells [73, 131, 132], results indicate an off-target effect of the ARF peptide [131]. Another recent study developed FOXM1 inhibitors by optimizing the potency of the best hits obtained from the screening of a large chemical compound library [130]. After several rounds of structure modifications, three compounds, NB-55, a monoamine analog, and NB-73 and NB-115, two more potent diammonium salts, were identified as FOXM1 inhibitors with desirable PK properties [130]. These compounds have been shown to affect FOXM1 activity by directly binding to it and enhancing its proteolytic degradation [130]. These compounds were found effective in inhibiting the growth of cancer cells in both cell lines and xenograft models [130, 133–135]. Unfortunately, these compounds also affect other FOX family members [135]; thus, further studies are required to check off-target effects.

**Table 6:** List of selected FOXM1 inhibitors.

S N	Inhibitors	Mechanisms	Cancers	Reference
1	Thiozole antibiotics (thiostrepton, siomycin A)	Proteasome inhibitors affect FOXM1 protein and RNA	Breast cancer, ovarian cancer, prostate cancer, acute lymphoblastic leukemia (ALL)	[124, 126, 127]
2	FDI-6	Blocks FOXM1 DBD, affect FOXM1-DNA interactions	Breast cancer, ovarian cancer, liver cancer, pancreatic cancer	[126, 128–130]
3	NB55, NB-73 and NB-115	Bind FOXM1, alter FOXM1 proteolytic sensitivity	Breast cancer, multiple myeloma, melanoma, high-grade serous ovarian cancer	[130, 133–135]
4	FOXM1-PROTAC	FOXM1 protein degradation	Breast cancer, Liver cancer	[129, 136, 137]
5	STL001	Relocalizes FOXM1 to the cytoplasm and promotes its autophagic degradation	Ovarian cancer, colorectal cancer, breast cancer, prostate cancer, esophageal cancer, and AML	[10, 27]

Proteolysis-targeting chimeras (PROTACs) are molecules that represent a promising strategy for targeted protein degradation. Recently, peptide-based FOXM1-PROTACs have also been developed [129, 136–138]. The novel FOXM1-PROTAC efficiently penetrates cancer cell membranes and induces the degradation of FOXM1 protein and thereby inhibits cancer cell proliferation and xenograft growth [136, 137]. FOXM1 may be a suitable target for peptide-based PROTAC degraders in cancer; however, further structural optimization studies will help to improve the potency and specificity. Our group has previously reported a novel compound, STL427944, a first-in-class small molecule inhibitor of FOXM1 [50]. STL427944 was discovered using a network-centric approach and confirmed as a selective FOXM1 inhibitor in various human cancer cells using RNAseq data [50]. We tested STL427944 in combination with different anticancer drugs; it sensitizes cancer cells of different etiology to multiple chemotherapies [50]. TL427944 has metabolic liabilities; it works at very high concentrations. To overcome these issues, we did structural activity relationship (SAR) optimization and found that the ring replacement in the structure of the parental compound STL427944 significantly improved the overall stability observed in its derivative, STL001 [10], and resulted in at least 10-fold more cellular-active compound with better “drug-like” properties [10]. STL427944 and its derivative STL001 affect FOXM1 activity in a two-step mechanism, first, it relocates nuclear FOXM1 to the cytoplasm, followed by autophagosomal degradation [10, 27, 50]. We found that STL001 treatment in cancer cells did not exert prominent cytotoxic action on its own, but sensitizes human cancers to a broad spectrum of cancer therapies, providing a rationale for combination therapy with these drugs [10, 27, 80]. Several FOXM1 inhibitors developed in the past are often non-specific and do not have “clinically” relevant potency (For a recent review, see [126]). Currently, there is no FOXM1 inhibitor available for clinical use. The FOXM1 inhibitors developed in recent years were found to be specific in the lab and have better activity than FDI-6. However, more preclinical work is still needed to evaluate their safety and efficacy in relevant animal models. Also, more studies are required to optimize drug delivery to maximize therapeutic efficacy and minimize side effects.

In recent years, several new strategies have been explored to develop selective FOXM1-targeted compounds, including the network-centric approach that was utilized in the development of FOXM1-selective small molecule inhibitor STL001 [50]. Another strategy based on interfering peptides disrupting protein–protein interactions was recently used to develop a FOXM1-targeting interfering peptide M1-20 [136]. Peptide proteolysis-targeting chimeras (p-PROTACs) based on FOXM1-PROTACs also represent a promising strategy for targeted protein degradation [136]. Aptamers, which are single-stranded RNA or DNA sequences obtained through the SELEX technique from random libraries, were also utilized recently to develop a FOXM1-specific single-stranded DNA aptamer (FOXM1 Apt) as an inhibitor of FOXM1 transcriptional functions in cancer cells [139]. Recently, nanoplatform-based strategies have drawn wide attention in drug delivery to enhance bioavailability, cellular permeability, and minimize off-target effects. The Nano-PROTAC prodrug NFTP, which is a self-assembled Peptide-PROTAC Prodrug targeting FOXM1, is a good example of a nanoplatform-based strategy [137]. Another good example, for dual-targeted delivery of Doxorubicin and FOXM1-Apt, is a DOX-Apts-HA-PEI-FOXM1 NPs, which are the FOXM1-Apt-Polyethylenimine nanoplatforms coated with Hyaluronic Acid and AS1411 Aptamer [140]. We and others have recently reported the potential of FOXM1 inhibitors in combination with different chemotherapeutic agents, BCL2 inhibitors, and cell cycle inhibitors, as discussed in more detail in the previous sections [10, 27, 80]. FOXM1 inhibitors synergize with several anticancer therapies. Hence, using FOXM1 inhibitors in combination with other anticancer drugs is also a good strategy to overcome therapy resistance. Today, these findings hold considerable promise for the expansion of translational studies and ultimately clinical trials with FOXM1 inhibitors in combination with other cancer therapies that may benefit cancer patients.

## Conclusion and future perspectives

Over the last decade, research targeting FOXM1 in cancer has accomplished significant progress; several small-molecule inhibitors (SMI) of FOXM1 have been identified and characterized (for a recent review, see [126]). FOXM1-dependent transcriptional activity promotes resistance to drug-induced senescence and apoptosis via regulating the expression of a plethora of genes involved in the genotoxic stress response, oxidative stress response, and mitotic catastrophe response. FOXM1 also controls different functions and pathways in drug response via directly interacting with other proteins. As discussed above, FOXM1 interacts with β-catenin and DVL2 and promotes their nuclear translocation. The above-described molecular mechanisms of drug resistance governed by FOXM1 open new scenarios for cancer therapy and hold promise for clinical benefits after targeting FOXM1 activity in cancer (Tables 1–3).

**Fig. 1:**
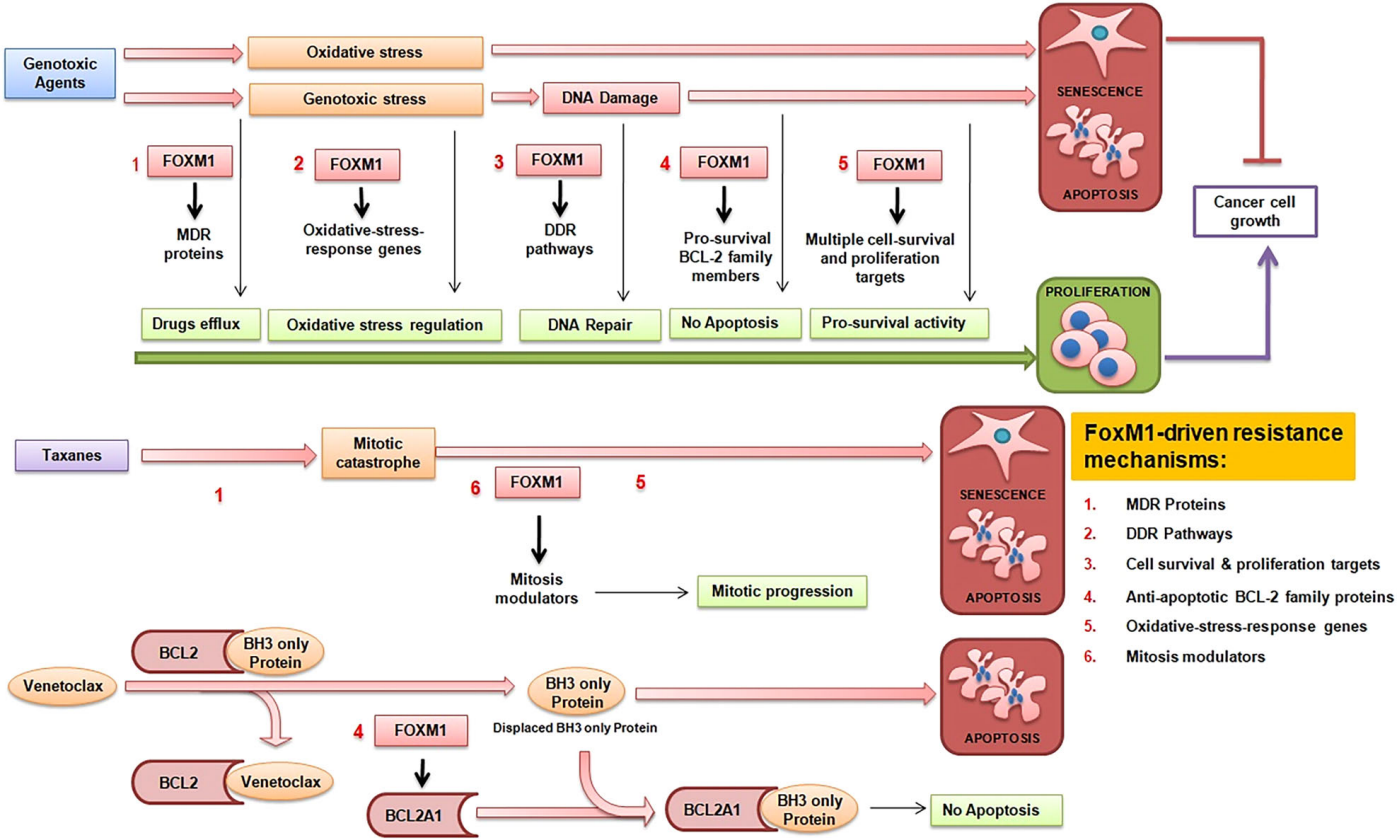
FOXM1-mediated mechanisms of resistance to apoptosis induced by various anticancer drugs.

FOXM1 activity in resistant cancer cells supports mechanisms that promote survival and evade senescence or apoptosis (Fig. 1). Its critical role in DNA damage repair after cells are exposed to DNA-damaging agents makes it an attractive therapeutic target in combination therapies with genotoxic drugs that render cancer cells dependent on DNA repair mechanisms. FOXM1 also creates a perfect axis for therapy resistance by modulating EMT and increasing drug-resistant CSCs, enhancing drug-efflux activity, regulating redox homeostasis, and increasing the anti-apoptotic BCL2-family proteins and other pro-survival pathways (Fig. 1). The above-described different functions of FOXM1 to escape senescence and apoptosis open new scenarios for advanced cancer therapies in which targeting of FOXM1 in combination with genotoxic drugs might be of clinical benefit. Indeed, several studies from our lab and others, already discussed above, have shown that targeting FOXM1 strongly sensitizes a variety of cancers to DNA-damage-induced senescence or apoptotic cell death (Tables 1–3).

The FOXM1 role in the suppression of taxane-induced senescence and apoptosis also offers possibilities of new therapeutic interventions. The taxanes are commonly used anti-mitotic chemotherapeutic agents; they induce mitotic arrest and apoptotic cell death by stabilizing microtubules. As discussed above, FOXM1 contributes to taxane resistance through diverse molecular mechanisms, such as increasing drug-efflux activity, UHRF1-controlled CSCs, increasing microtubule dynamics modulators and different pro-survival pathways (Table 4, Fig. 1). The functions of FOXM1 in taxane resistance are different from those involved in the resistance to genotoxic agents; however, we and others have identified that pharmacological targeting of FOXM1 synergized with microtubule inhibitors and induced senescence and apoptotic cell death (Table 4). These studies have validated FOXM1 as a potential therapeutic target in combination therapy to overcome taxane resistance.

Several studies, discussed above, have also described the functions of FOXM1 in the resistance to different targeted therapies. The mechanisms are poorly defined; however, FOXM1 inhibitors combined with different molecular-targeted therapies have been recognized with great synergy for cancer treatment. Therefore, there is a strong rationale for combining pharmacological inhibitors of FOXM1 with targeted therapy. Recently, already described above, we have investigated that FOXM1 creates a perfect axis for resistance to venetoclax-induced apoptosis by increasing the anti-apoptotic BCL2 family protein, BCL2A1, effectively protecting AML cells from venetoclax-induced apoptosis. Therefore, we proposed pharmacological targeting of FOXM1 using STL001 with venetoclax to overcome resistance to venetoclax-induced apoptosis in AML (Table 5, Fig. 1).

TF are conventionally considered difficult to therapeutically target. Therefore, being a TF, its “druggability” is currently one of the limitations for FOXM1 inhibitor-based therapies in cancer. Currently, no FOXM1 inhibitor has advanced to clinical trials. However, data from preclinical studies have shown the future possibility for FOXM1 inhibitors as a component of combination therapy [126]. Abnormally high expression levels of FOXM1 in cancer cells compared to normal differentiated cells make it an attractive target for therapies. The Human Protein Atlas (HPA) data show FOXM1 expression in normal tissues with high cell proliferation, such as bone marrow, testis, and thymus, although at levels considerably lower than in cancer cells. In addition, FOXM1 activity can be detected during regenerative processes initiated after injury in tissues, such as the lung and liver [141, 142]. Therefore, the potential side effects of FOXM1 suppression in cancer patients are likely to be limited, but might impair normal functions of some tissues and regeneration/healing of injured tissues [143]. Recent reports based on in vivo studies found FOXM1 inhibitors effective in suppressing FOXM1 activity and eliminating cancer cells with minimal effects on normal cells [144, 145]. In the recent past, several different small molecules have been identified and characterized as FOXM1 inhibitors (for a recent review, see [126]). While some of the newly discovered inhibitors of FOXM1 are specific in the lab, further molecular studies are required to explore the precise mechanism of their action, and extensive pre-clinical work is also needed to ensure specificity and safety. This will help to develop the SMI of FOXM1 for cancer treatment.

In conclusion, several studies discussed in this review have identified that FOXM1 inhibitors have great synergy with different traditional chemotherapies and targeted cancer therapies. In the current time, the research on FOXM1 inhibitors is expanding because FOXM1 plays a unique role in different aspects of tumor development and progression by modulating drug sensitivity and resistance. This supports the future possibility of FOXM1 inhibitors working best in combination with different classes of cancer drugs to overcome therapy resistance in cancer cells.
